# Identify predictive factors for the emergence of self-reported oropharyngeal dysphagia in older men and women populations: a retrospective cohort analysis

**DOI:** 10.3389/fneur.2025.1439579

**Published:** 2025-02-21

**Authors:** Shuang Liu, Ling-Jie Fan, Hao Tian, Sha-Sha Wei, Si-Jie Zhang, Mei He, Ji-Hong Wei

**Affiliations:** ^1^Mianyang Central Hospital, School of Medicine, University of Electronic Science and Technology of China, Mianyang, China; ^2^Chengdu Medical College, Chengdu, China; ^3^College of Computer Science, Sichuan University, Chengdu, China; ^4^School of Communication and Information Engineering, Chongqing University of Posts and Telecommunications, Chongqing, China

**Keywords:** oropharyngeal dysphagia, gender difference, machine learning, older adult, ML

## Abstract

**Background and objectives:**

Oropharyngeal dysphagia (OD) is an emergent health concern in older adults, with incidence rates escalating due to age-related and various neurological and physical conditions. This study identifies risk and protective factors for new-onset OD, with an emphasis on gender differences.

**Methods:**

Utilizing data from the National Health and Aging Trends Study (NHATS), this study analyzed 6,360 participants (58.1% women) across 2011–2014 and 2015–2018 periods. Employing a random forest feature selection, specifically recursive feature elimination and mean decrease impurity algorithm, we assessed 128 variables to identify critical factors including demographics, health, physical and neurological functionality, and environmental conditions. The study further applied logistic regression and explored factor interactions using restricted cubic splines, streamlining the analysis to focus on key determinants of oropharyngeal dysphagia.

**Results:**

Initial findings show a decrease in new-onset OD from 15.62% in 2011 to 14.49% in 2015, with women more frequently affected. The analysis elucidates a constellation of highly predictive factors for OD, encompassing extremes of body mass index (BMI), socioeconomic challenges (as indicated by low income), diminished physical conditioning, and adverse emotional states. Notably, gender-specific disparities emerged, highlighting the critical role of cognitive function and mood in men, whereas in women, the overarching influence of general health status and comorbidities was more pronounced.

**Conclusion:**

This condensed examination highlights the complex, multifactorial nature of OD in older adults, influenced by sociodemographic, physical, and psychological factors, and underscores the need for gender-specific approaches in predicting, preventing, and managing OD.

## Introduction

In the domain of geriatric healthcare, oropharyngeal dysphagia (OD) emerges as a paramount concern, with its prevalence intricately linked to the demographic shifts toward an older population. Scholarly investigations reveal a notable escalation in the incidence of OD, with the prevalence rates among older adults ranging from 10 to 33%, as documented in seminal studies (1, 2). This prevalence is alarmingly higher, oscillating between 8.1 and 80%, within cohorts suffering from neurological impairments or conditions, including but not limited to cerebrovascular accidents, Alzheimer’s disease, and Parkinson’s disease (3, 4). The ramifications of oropharyngeal dysphagia on the health and well-being of the aging population are profound and multifarious. The condition is a harbinger of grave health complications such as malnutrition (5), dehydration (6), and aspiration pneumonia (7), each significantly exacerbating the mortality risk associated with the affected demographics (8, 9). Moreover, the impact of OD transcends the physical domain, casting a long shadow over the mental health and overall quality of life of those afflicted (10, 11).

Within the United States, the economic repercussions of OD are profound, with estimations suggesting an annual fiscal impact exceeding $7 billion due to both direct and indirect costs (12). Notably, individuals hospitalized with OD face an augmented inpatient expense, exceeding those without OD by approximately $4,282 annually (13), underscoring its considerable contribution to global disability. The societal and economic ramifications of OD are poised for amplification in tandem with escalating healthcare costs and the anticipated surge in the demographic segment aged 60 and above (14), expected to rise from 12 to 22% by the year 2050 (15). Consequently, the formulation of cost-effective and efficacious intervention methodologies emerges as imperative, necessitating a comprehensive understanding of the myriad risk factors for OD within the aging population.

Extant literature delineates a variety of factors implicated in the etiology of OD, ranging from neurological afflictions (16, 17) and physical trauma (18) to the impacts of certain medications (19). Moreover, OD’s association with individual characteristics—such as age (20), obesity (21), lifestyle habits like smoking (22), and even emotional and partner employment status (23)—underscores the intricate web of determinants influencing its development. This intricacy mandates a sophisticated and multifaceted research approach to elucidate the nuances of OD in aging individuals, thereby refining prevention and management paradigms for this prevalent disorder.

A particularly promising avenue for exploration resides in the gender-specific aspects of oropharyngeal dysphagia. Initial investigations reveal marked disparities in the incidence and clinical manifestation of OD between men and women cohorts (24), hinting at the potential for gender-differentiated protective and risk factors. This observation suggests a requisite for bespoke research methodologies and clinical interventions. The etiological complexity of oropharyngeal dysphagia challenges conventional research modalities, with the majority of studies adopting cross-sectional designs (25–27). The reliance on subjective criteria for feature selection in traditional approaches hampers the exhaustive examination of the multifactorial relationships between potential predictors and OD incidence, particularly in analyses incorporating a broad array of predictor variables.

In light of these considerations and acknowledging the absence of comprehensive comparative analyses of sociodemographic, health, cognitive, and functional predictors of dysphagia risk with a focus on gender disparities, our study endeavors to bridge this knowledge gap. By leveraging a vast publicly accessible dataset and integrating traditional statistical techniques with advanced machine learning algorithms, we aim to dissect the occurrence of OD through a gender-stratified lens, assessing 128 potential risk and protective factors to illuminate the path forward in understanding and combating this condition.

## Methods

This study is characterized as a retrospective cohort analysis, underpinned by the utilization of data from the National Health and Aging Trends Study (NHATS). NHATS stands as a publicly accessible, nationally representative longitudinal survey specifically targeting Medicare beneficiaries aged 65 and older residing within the United States. Its primary objective is to delineate the risk factors associated with late-life disability among the aging demographic, thereby augmenting the corpus of knowledge pertaining to the trajectories of functional decline and resilience observed in older adults (28). Initiated in 2011, the initial cohort has been subjected to annual follow-up assessments, facilitating a comprehensive longitudinal examination of the dynamic changes and continuities experienced by this population over time.

### Participants

We analyzed data across eight rounds of NHATS, spanning from 2011 through 2018. This encompassed an extensive review of participant data commencing with their initial interview and extending through the subsequent 3 years, offering a longitudinal perspective on their health trajectories. Within the baseline years of 2011 and 2015, a total of 12,437 NHATS participants engaged in the survey. From this initial cohort, exclusions were applied judiciously to ensure the integrity of the study’s focus on new-onset OD. Specifically, 30 participants (20 in 2011 and 10 in 2015) were excluded due to the absence of baseline interviews pertaining to swallowing disorders. Furthermore, an additional 630 participants (400 in 2011 and 230 in 2015) were excluded for having pre-existing swallowing disorders at baseline. The meticulous exclusion criteria extended to 4,988 participants (3,508 between 2012 and 2014 and 1,480 between 2016 and 2018) who were unable to complete follow-up interviews, alongside 406 individuals who failed to provide specific OD outcomes in the subsequent three-year periods. An additional 30 participants were excluded due to having more than 20% missing data on independent variables. This rigorous selection process culminated in a refined final sample of 6,360 participants, of whom 58.1% were women.

### Measures

#### New-onset of OD

OD is typically defined as difficulty in the process of initiating a swallow and moving food or liquid from the mouth to the esophagus. In this study, we operationalized new-onset OD based on self-reported difficulties with chewing or swallowing. Participants were systematically queried during the follow-up phases (2012–2014 and 2016–2018) regarding their recent experiences with mastication or deglutition difficulties, specifically inquiring, “In the past month, have you experienced any chewing or swallowing problems leading to difficulty eating?” Responses were binary, encapsulating either an affirmative or negative experience. New-onset dysphagia was rigorously defined by the absence of such swallowing difficulties at the initial survey point (either 2011 or 2015) juxtaposed with the emergence of dysphagia within the subsequent 36-month observational window.

#### Demographics and daily activity

The demographic canvas included an array of variables such as age, stature, mass, body mass index (BMI), ethnoracial identity, educational attainment, income bracket, domestic arrangements (solo living or cohabitation with a spouse/partner), familial size, and progeny count, as recorded in the years 2011 and 2015. Lifestyle inquiries probed smoking habits, manual dominance, preferred pastimes, frequency of entertainment engagements in the preceding month, participation in ambulatory exercises, valuation of outdoor ventures, and proficiency in activities of daily living (e.g., transitional movements, ambulation, attire management, personal hygiene, and lavatory use).

#### Health condition

The health dimension encompassed self-assessments of overall health and detailed medical histories inclusive of cardiovascular incidents, heart conditions, hypertension, arthritic conditions, osteoporosis, diabetes mellitus, pulmonary diseases, cerebrovascular accidents, cognitive degeneration, oncological diagnoses, and skeletal fractures, alongside a tally of comorbid conditions. This was supplemented with details on surgical interventions, pain experiences, functional limitations of the appendages, and sensory impairments affecting vision and audition, in addition to sleep-related disorders.

#### Physical and neurological function

Measures of physical functioning included recording participants’ total and sub-scores for performing the three tasks of the SPPS (Short Physical Performance Battery) including Balance Test, Walking Test and Repeat Chair Test (29). Additional inclusion was made of participants’ one-legged eyes-open/eyes-closed standing test scores, mean vs. best grip/breath test scores, and self-reported walking ability (whether they could walk three blocks/ten steps).

Neurological functioning recorded participants’ cognitive functioning as well as mood status. Among the measures of cognitive functioning were participants’ self-reported memory status, Clock Drawing Test scores and Immediate/Delayed Word Recall Test scores, and also Date Recall and Presidential Recall Tests. Mood status was screened for the presence of depression/anxiety symptoms in participants using the PHQ4 (30).

#### Environment condition

Recognizing the potential influence of adverse living conditions on swallowing disorders, an examination of the participants’ residential and surrounding environments was conducted. This entailed an inspection of dwelling and entryway structures (e.g., stairs, ramps), interior and exterior safety risks (e.g., structural integrities, neighborhood cleanliness, and security concerns), and bathroom configurations, aiming to establish a correlation with the prevalence of OD.

#### Data analysis

The analysis commenced with the initial processing of data and the application of fundamental statistical methodologies, including T-tests, via the SPSS platform. The subsequent analysis into a more sophisticated computational realm, utilizing the Python programming environment within PyCharm (version 2022.1.3) and leveraging the advanced capabilities of the scikit-learn library (version 1.3.2) for feature selection, recursive feature elimination (RFE), and the construction and nuanced interpretation of both univariate and multivariate analytical models.

In 2011, the prevalence of OD initiation within a triennial span was quantified at 15.62%, whilst a slight diminution was observed in 2015, with the incidence rate adjusting to 14.49%. A gender-stratified analysis revealed a discernible disparity, with women exhibiting a higher incidence rate of 15.8% in comparison to men at 14.38%. The median age bracket of participants situated between 75 and 79 years and the racial composition was predominantly non-Hispanic white, comprising 70.9% (4,458 individuals) of the sample cohort (Table 1).

**Table 1:** Demographic information stratified by gender.

Feature		Men (*N* = 2,664)		Women (*N* = 3,696)	
		Onset of OD	None-OD	Onset of OD	None-OD
Age	65–69	86(12.78%)	587(87.22%)	112(14.27%)	673(85.73%)
	70–74	75(12.36%)	532(87.64%)	88(11.62%)	669(88.38%)
	75–79	75(13.91%)	464(86.09%)	115(15.35%)	634(84.65%)
	80–84	59(12.58%)	410(87.42%)	113(16.07%)	590(83.93%)
	85–89	59(22.43%)	204(77.57%)	81(19.66%)	331(80.34%)
	90-	29(25.66%)	84(74.34%)	75(25.86%)	215(74.14%)
Education	Below high school	195(16.64%)	977(83.36%)	341(17.93%)	1,561(82.07%)
	Above high school	188(12.6%)	1,304(87.4%)	243(13.55%)	1,551(86.45%)
Living arrangement	Alone	84(14.63%)	490(85.37%)	242(15.57%)	1,312(84.43%)
	With partner	202(13.27%)	1,320(86.73%)	134(11.72%)	1,009(88.28%)
	With partner and others	52(15.29%)	288(84.71%)	35(16.36%)	179(83.64%)
	With others only	45(19.74%)	183(80.26%)	173(22.04%)	612(77.96%)
Height		1.74(0.11)	1.76(0.11)	1.63(0.09)	1.64(0.10)
Weight		83.70(16.81)	87.31(16.71)	72.91(17.59)	72.432(16.36)
BMI		27.71(6.16)	28.56(6.40)	27.53(6.94)	27.23(6.65)
Income		50,664(46091)	70,599(61055)	33,688(32770)	45,114(48,350)
Kid number		0.21(0.56)	0.17(0.53)	0.34(0.60)	0.23(0.58)
Smoke regularly	Yes	250(14.73%)	1,447(85.27%)	216(14.89%)	1,235(85.11%)
	No	133(13.75%)	834(86.25%)	368(16.39%)	1877(83.61%)
Disease sum		2.63(1.63)	2.21(1.48)	3.09(1.59)	2.47(1.46)
Visual impairment	Yes	80(15.63%)	432(84.38%)	141(18.63%)	616(81.37%)
	No	303(14.08%)	1849(85.92%)	443(15.07%)	2,496(84.93%)
Hearing impairment	Yes	78(17.14%)	377(82.86%)	80(19.85%)	323(80.15%)
	No	305(13.81%)	1904(86.19%)	504(15.31%)	2,789(84.69%)
Health status	Excellent	38(9.11%)	379(90.89%)	44(8.76%)	458(91.24%)
	Very good	104(12.46%)	731(87.54%)	132(11.72%)	994(88.28%)
	Good	127(14.65%)	740(85.35%)	191(15.37%)	1,052(84.63%)
	Fair	83(19.21%)	349(80.79%)	165(25.27%)	488(74.73%)
	Poor	31(27.43%)	82(72.57%)	52(30.23%)	120(69.77%)

Gender stratification of the dataset served as the initial step, allowing for the nuanced examination of categorical and continuous variables, presented as counts with percentages and means with standard deviations, respectively. The exclusion of individuals presenting with more than 20% missing data in crucial variables ensured the analytical purity of the study. The remaining gaps in the dataset were adeptly bridged via the iterative imputation prowess of the Missforest algorithm, thereby preserving the dataset’s completeness for subsequent analysis. The process began by separating categorical and numerical variables, with categorical variables undergoing one-hot encoding to facilitate imputation and numerical variables were processed directly. A variable type indicator was created to distinguish between continuous (0) and categorical (1) variables, ensuring appropriate treatment during imputation. The MissForest algorithm was then applied with the following parameters: maximum iterations set to 10, number of trees in each forest set to 100, and all available processor cores utilized. Post-imputation, one-hot encoded categorical variables were converted back to their original form by selecting the category with the highest probability.

The random forest methodology, a machine learning algorithm that constructs and merges multiple decision trees for more accurate and stable predictions, was employed in our study to select the most important predictors of oropharyngeal dysphagia (OD) from an initial pool of 128 candidate variables. We specifically utilized the random forest feature recursive elimination (RFE) method, which iteratively constructs models, evaluates feature importance, and removes less significant features until reaching the desired number. To ensure the robustness and stability of our feature selection process, we implemented a cross-validation approach. Specifically, we used the GridSearchCV function from scikit-learn with 5-fold cross-validation to optimize the random forest parameters. The hyperparameters tuned included the number of estimators (100, 200, 300), maximum depth (None, 5, 10), and minimum samples split (2, 5, 10) (31). This cross-validation process not only helped in selecting stable features but also in identifying the optimal model parameters. The RFE process was set to select the top 20 features. To further refine our variable selection, we incorporated the Mean Decrease Impurity (MDI) algorithm, an integral component of the random forest methodology. This step quantified each feature’s ability to reduce impurity, providing an empirical basis for their inclusion in the predictive model.

Subsequently, we conducted univariate logistic regression analyses to elucidate the relationship between these chosen variables and the development of OD. Variables demonstrating statistical significance (*p* < 0.05) were then incorporated into a multivariate logistic regression model. This final phase allowed us to delineate the statistical significance and potential predictive utility of these variables through calculated *p*-values and confidence intervals. To address potential multicollinearity issues, we performed a Variance Inflation Factor (VIF) analysis on the selected variables. The VIF matrix were calculated to assess the degree of correlation between predictors. The results of this analysis are presented in Supplementary Tables S1, S2.

Moreover, the incorporation of Restricted Cubic Splines (RCS) facilitated the intricate analysis of non-linear relationships between variables and the onset of oropharyngeal dysphagia. This advanced statistical technique provided a nuanced lens through which the complex interplay of predictive factors could be interpreted, significantly enhancing the study’s analytical rigor and the interpretability of its findings.

## Results

**Figure 1:**
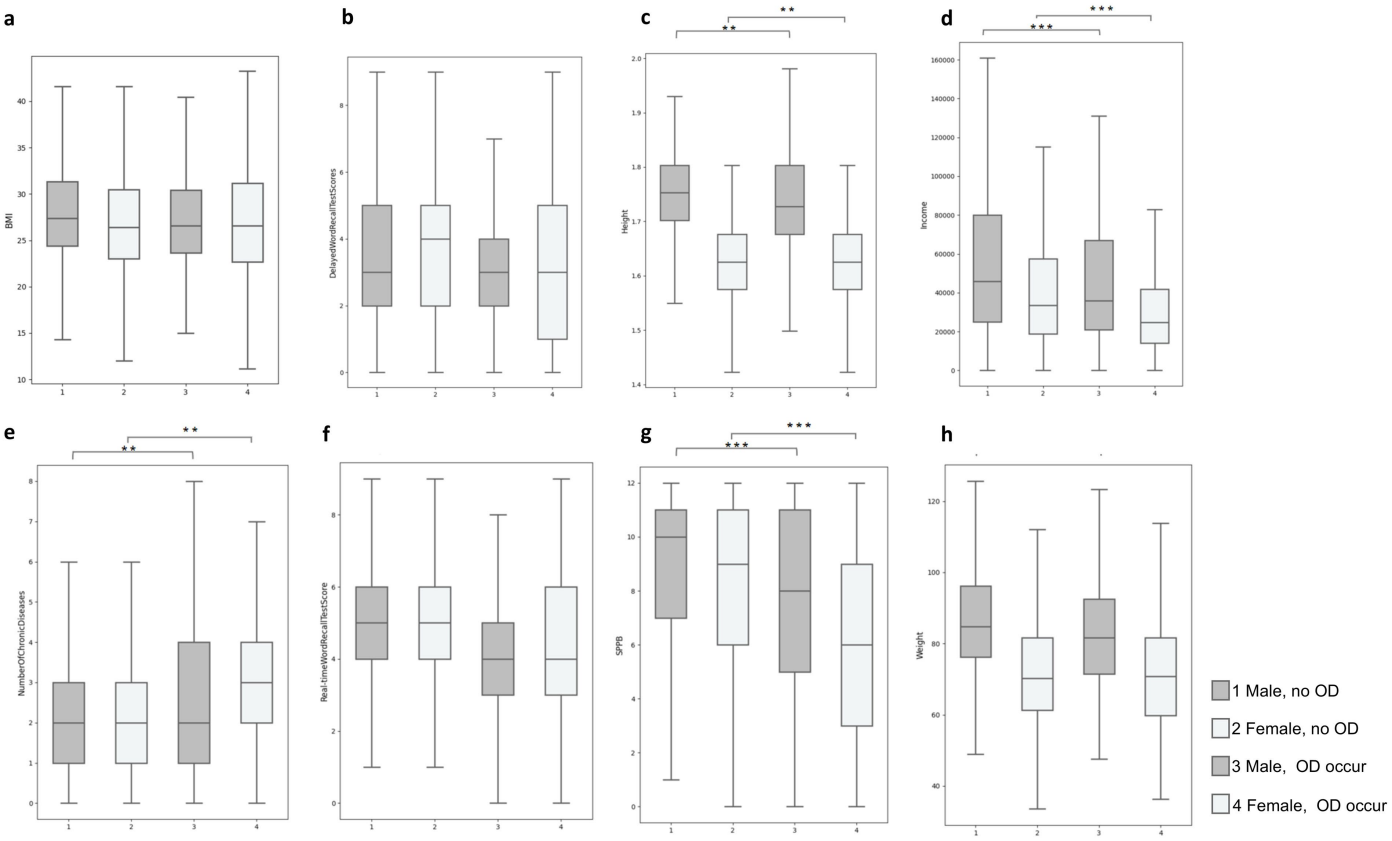
Quartile plots of the distribution of differences in typical characteristics of occurring and non-occurring OD in male versus female populations: **(A)** BMI, **(B)** Delay words recall test scores, **(C)** Height, **(D)** Income, **(E)** Number of chronic diseases, **(F)** Real time words recall test scores, **(G)** SPPB score, **(H)** Height **p < 0.01; ***p < 0.001.

Analytical comparisons conducted via T-tests illuminated significant contrasts between individuals with and without one-set OD. Notably, those afflicted with OD demonstrated lower educational levels (men: 16.64% without one-set OD vs. 12.6% one-set OD; women: 17.93% without one-set OD vs. 13.55% one-set OD), diminished average income (men: $70,599.21 without one-set OD vs. $50,662.94 one-set OD; women: $45,114.28 without one-set OD vs. $33,688.12 with one-set OD), and a heightened burden of comorbidities (men: 2.21 without one-set OD vs. 2.63 one-set OD; women: 2.47 without one-set OD vs. 3.09 one-set OD) as delineated in Figure 1.

**Figure 2:**
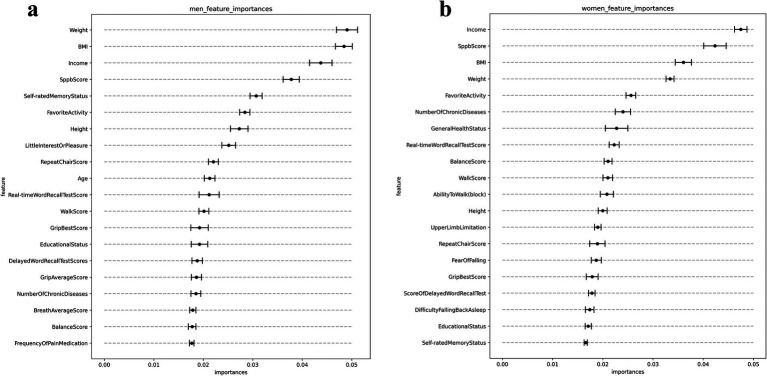
Top 20 features filtered by RFE and MDI calculations. **(A)** Top 20 features for men, **(B)** top 20 features for women. SPPB score: Short Physical Performance Battery score (0–12), assessing lower extremity function through balance, gait speed, and chair stand tests; Delayed Word Recall Test Scores: Number of words correctly recalled after a delay, from a list of 10 words presented earlier. Real-time Word Recall Test Score: Number of words immediately recalled from a list of 10 words. Grip Best Score: Highest recorded grip strength measurement using a dynamometer, best of two trials per hand. Grip Average Score: Average grip strength (kg) across all trials. Repeat Chair Score: Time to complete five consecutive chair stands without using arms (0–4). Walk Score: Time to walk 3 meters at usual pace (0–4). Balance Score: Ability to maintain balance in side-by-side, semi-tandem, and full tandem positions (0–4). Breath Average Score: Peak expiratory flow rate (L/min), average of two measurements using a peak flow meter.

Subsequent to the execution of 10 iterations of RFE averaged for analytical rigor, Figures 2A,B elucidate the principal 20 variables forecasting OD in older adults of both genders, adjudged by the MDI criterion. This nuanced analysis, with exhaustive rankings available in Supplementary Table S1, showcased a substantial overlap in the key predictive variables across genders. For the demographics, “weight” emerged as the paramount predictor, succeeded by “BMI” and “income.” Conversely, for women, “income” was identified as the foremost predictor, followed by scores on the Short Physical Performance Battery (SPPB) and “BMI.” Within the intermediary tier of predictors (ranks 4–10), physical performance metrics notably influenced OD risk for both genders. However, the men group also highlighted cognitive and emotional dimensions—specifically “self-reported memory status” and “lack of interest or pleasure”—as lower-contribution predictors.

In alignment with the RFE analysis outcomes, the subsequent univariate analyses across both genders delineated a notable association between economic status and cognitive self-assessment with the incidence of OD. Specifically, among male participants, the odds ratio (OR) for economic status was precisely 1.000 [95% Confidence Interval (CI): 1.000–1.000, *p* < 0.001], while the OR for self-reported cognitive status stood at 1.467 (95% CI: 1.313–1.638, *p* < 0.001). Correspondingly, for female participants, the OR for economic status mirrored that of their male counterparts at 1.000 (95% CI: 1.000–1.000, *p* < 0.001), and the OR for cognitive self-assessment was observed at 1.384 (95% CI: 1.258–1.522, *p* < 0.001). The VIF matrix were calculated to assess the degree of correlation between predictors. The results of this analysis are presented in Supplementary Tables S2, S3.

In the men cohort, stature was significantly correlated with one-set OD in univariate analysis (OR: 0.364, 95% CI: 0.136–0.974, *p* = 0.044); however, this correlation dissipated in the multivariate analysis (OR: 0.201, 95% CI: 0.003–13.034, *p* = 0.451). Conversely, variables pertaining to self-rated cognitive performance and anhedonia retained their significance in multivariate analysis, suggesting a profound linkage with one-set OD manifestation (self-rated memory status OR: 1.266, 95% CI: 1.123–1.427, *p* < 0.001; anhedonia OR: 1.261, 95% CI: 1.120–1.421, *p* < 0.001). For the women, the physical capacity to traverse three city blocks exhibited a significant univariate correlation (OR: 2.503, 95% CI: 2.092–2.995, *p* < 0.001), which did not persevere in multivariate analysis (OR: 1.179, 95% CI: 0.924–1.504, *p* = 0.185). Nonetheless, the apprehension regarding potential falls persisted as a significant multivariate correlate (OR: 0.693, 95% CI: 0.567–0.846, *p* < 0.001), evidencing a robust, independent influence on OD incidence (refer to Tables 2, 3).

**Table 2:** Results of univariate logistic regression tests and multivariate logistic regression tests on the occurrence of OD in men.

Variable	OR	95% CI	OR	95% CI	*p*-value
	Univariate		Multivariate		
Height	0.364	(0.136, 0.974)	0.201	(0.003, 13.034)	0.451
Weight	0.986	(0.979, 0.993)	0.997	(0.957, 1.040)	0.903
BMI	0.977	(0.959, 0.995)	0.966	(0.855, 1.092)	0.582
Income	1.000	(1.000, 1.000)	1.000	(1.000, 1.000)	0.155
Sppbscore	0.894	(0.868, 0.921)	0.928	(0.835, 1.033)	0.172
DelayedWordRecallTestScores	0.921	(0.870, 0.975)	1.109	(1.015, 1.211)	**0.023**
Real-timeWordRecallTestScore	0.843	(0.788, 0.902)	0.907	(0.815, 1.008)	0.071
GripBestScore	0.797	(0.734, 0.866)	0.800	(0.498, 1.284)	0.354
GripAverageScore	0.800	(0.737, 0.869)	1.210	(0.752, 1.946)	0.432
RepeatChairScore	0.773	(0.713, 0.839)	0.990	(0.842, 1.164)	0.902
WalkScore	0.770	(0.705, 0.840)	1.035	(0.879, 1.217)	0.682
BalanceScore	0.774	(0.710, 0.844)	1.013	(0.862, 1.189)	0.879
Age	1.159	(1.078, 1.246)	1.029	(0.943, 1.123)	0.517
NumberOfChronicDiseases	1.194	(1.114, 1.280)	1.079	(0.999, 1.166)	0.053
FrequencyOfPainMedication	0.847	(0.790, 0.909)	0.900	(0.835, 0.971)	**0.006**
EducationalStatus	0.928	(0.887, 0.972)	1.024	(0.967, 1.084)	0.421
BreathAverageScore	0.797	(0.727, 0.873)	1.021	(0.908, 1.149)	0.729
Self-ratedMemoryStatus	1.467	(1.313, 1.638)	1.266	(1.123, 1.427)	***P* < 0.001**
LittleInterestOrPleasure	1.413	(1.267, 1.576)	1.261	(1.120, 1.421)	***P* < 0.001**

SPPB score: Short Physical Performance Battery score (0–12), assessing lower extremity function through balance, gait speed, and chair stand tests; Delayed Word Recall Test Scores: Number of words correctly recalled after a delay, from a list of 10 words presented earlier. Real-time Word Recall Test Score: Number of words immediately recalled from a list of 10 words. Grip Best Score: Highest recorded grip strength measurement using a dynamometer, best of two trials per hand. Grip Average Score: Average grip strength (kg) across all trials. Repeat Chair Score: Time to complete five consecutive chair stands without using arms (0–4). Walk Score: Time to walk 3 meters at usual pace (0–4). Balance Score: Ability to maintain balance in side-by-side, semitandem, and full tandem positions (0–4). Breath Average Score: Peak expiratory flow rate (L/min), average of two measurements using a peak flow meter. Bold values indicate *p* < 0.05.

**Table 3:** Results of univariate logistic regression tests and multivariate logistic regression tests on the occurrence of OD in women.

Variable	OR	95% CI	OR	95% CI	*p*-value
	Univariate		Multivariate		
SppbScore	0.880	(0.860, 0.900)	0.947	(0.866, 1.035)	0.227
Income	1.000	(1.000, 1.000)	1.000	(1.000, 1.000)	0.056
NumberOfChronicDiseases	1.307	(1.233, 1.384)	1.108	(1.035, 1.185)	0.003
Real-timeWordRecallTestScore	0.814	(0.773, 0.857)	0.851	(0.784, 0.923)	***P* < 0.001**
UpperLimbLimitation	0.435	(0.363, 0.522)	0.716	(0.582, 0.881)	**0.002**
BalanceScore	0.710	(0.659, 0.764)	0.999	(0.862, 1.158)	0.989
WalkScore	0.690	(0.639, 0.745)	0.981	(0.852, 1.131)	0.796
RepeatChairScore	0.742	(0.694, 0.794)	1.077	(0.938, 1.235)	0.292
DelayedWordRecallTestScores	0.910	(0.873, 0.948)	1.088	(1.018, 1.162)	**0.012**
GripBestScore	0.672	(0.610, 0.741)	0.890	(0.797, 0.993)	**0.038**
GeneralHealthStatus	1.517	(1.393, 1.652)	1.073	(0.963, 1.196)	0.204
EducationalStatus	0.896	(0.857, 0.936)	1.015	(0.962, 1.072)	0.585
AbilityToWalk(block)	2.503	(2.092, 2.995)	1.179	(0.924, 1.504)	0.185
DifficultyFallingBackAsleep	0.834	(0.776, 0.897)	0.920	(0.854, 0.991)	**0.028**
FearOfFalling	0.449	(0.375, 0.538)	0.693	(0.567, 0.846)	***P* < 0.001**
Self-ratedMemoryStatus	1.384	(1.258, 1.522)	1.080	(0.971, 1.201)	0.157

*SPPB score: Short Physical Performance Battery score (0–12), assessing lower extremity function through balance, gait speed, and chair stand tests. Real-time Word Recall Test Score: Number of words immediately recalled from a list of 10 words. Balance Score: Ability to maintain balance in side-by-side, semi-tandem, and full tandem positions (0–4). Repeat Chair Score: Time to complete five consecutive chair stands without using arms (0–4). Walk Score: Time to walk 3 meters at usual pace (0–4). Delayed Word Recall Test Scores: Number of words correctly recalled after a delay, from a list of 10 words presented earlier. Grip Best Score: Highest recorded grip strength measurement using a dynamometer, best of two trials per hand. Bold values indicate *p* < 0.05.

**Figure 3:**
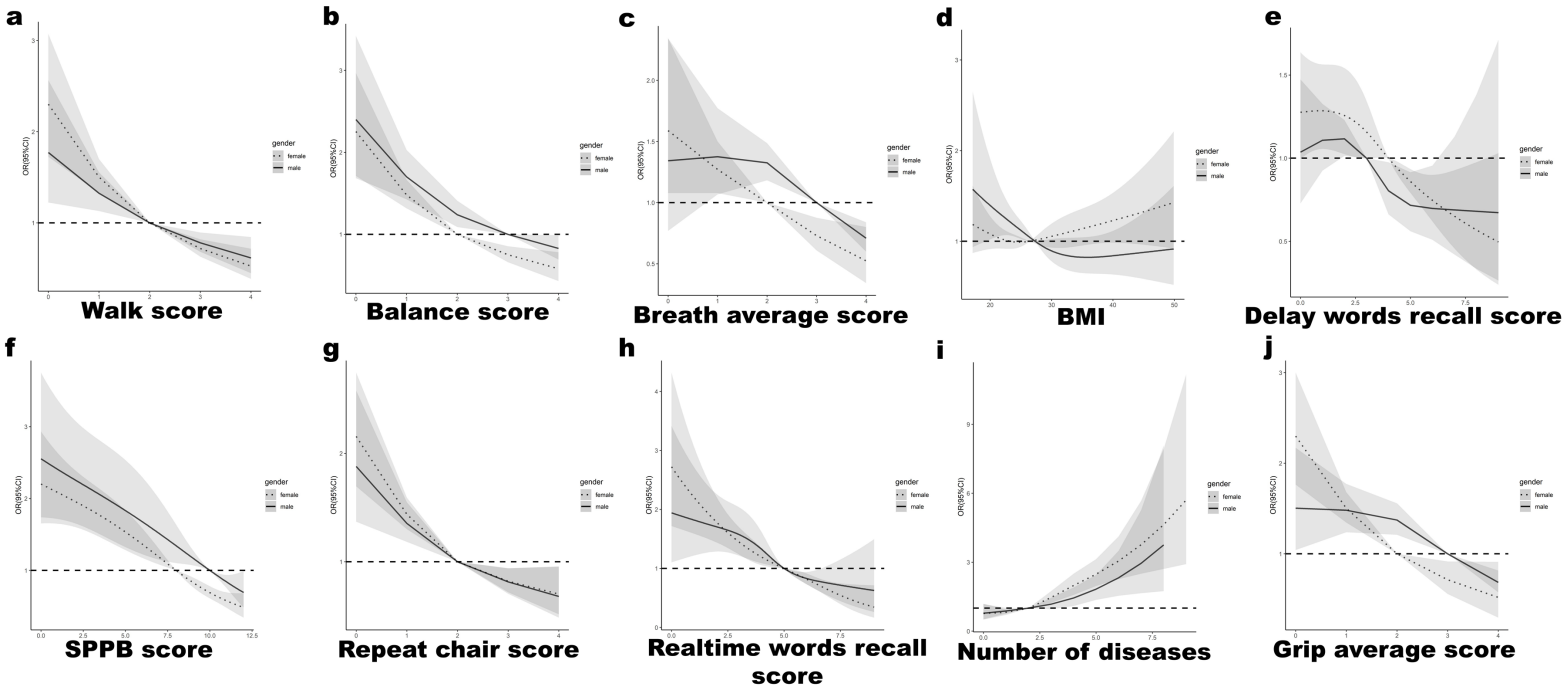
RCS curve analysis for male vs. female for top characteristics: **(A)** Walk score, **(B)** Balance score, **(C)** Breath average score, **(D)** BMI, **(E)** Delay words recall test scores, **(F)** SPPB score, **(G)** Repeat chair score, **(H)** Real time words recall test scores, **(I)** Number of chronic diseases, **(J)** Grip average score.

RCS analysis underscored that superior physical fitness parameters, such as SPPB scores, grip strength, and respiratory function, inversely correlated with dysphagia risk (illustrated in Figures 3A–C). Similarly, enhancements in cognitive faculties were linked with diminished dysphagia occurrence (depicted in Figure 3D). Moreover, an escalation in chronic disease count was directly proportional to increased NI (Figure 3E). Notably, both the lower and upper extremes of Body Mass Index (BMI) were implicated in elevated dysphagia risk (Figure 3F). The risk of OD decreased as the repeat chair score, realtime words recall score, and Grip average score increased (Figures 3G, H, J). As the number of chronic diseases increased, their risk of OD increased (Figure 3I).

## Discussion

This retrospective cohort investigation employed a comprehensive analytical approach to elucidate the relative contribution of 128 potential protective and risk factors in the pathogenesis of one-set OD. We utilized a synthesis of Random Forest based RFE and MDI analysis to identify the most significant predictors. Subsequently, we applied both univariate and multivariate logistic regression modeling techniques to quantify the associations between these predictors and OD incidence.

Our findings revealed a notable prevalence of new-onset OD within a three-year span, with rates of 15.62% in 2011 and 14.49% in 2015. This slight decrease over time may reflect improvements in healthcare practices or increased awareness of OD prevention strategies. Importantly, we observed a gender disparity in OD prevalence, with women exhibiting a higher rate (15.8%) compared to men (14.38%).

The observed gender disparities in OD prevalence and associated risk factors reflect a complex interplay of biological, physiological, and sociocultural factors, underscoring the need for gender-specific approaches in OD prevention and management. Our comprehensive analysis reveals distinct patterns of risk factors between men and women, highlighting the importance of tailored interventions. In women, we found a stronger association between comorbidities and OD risk, which may be attributed to physiological differences such as lower muscle mass, especially in the oropharyngeal region, and a higher comorbidity burden including conditions like osteoporosis and autoimmune disorders that can indirectly impact swallowing mechanics. Additionally, women’s increased susceptibility to malnutrition and vitamin deficiencies in older age groups may further contribute to their OD risk. Conversely, in men, our analysis revealed a more pronounced correlation between psychological factors, particularly anhedonia, and OD risk. This gender-specific pattern may be explained by neurobiological differences in stress responses, the impact of age-related testosterone decline on both muscle maintenance and mood regulation, and sociocultural factors such as traditional masculinity norms that may delay healthcare-seeking behaviors. Furthermore, men’s higher likelihood of engaging in behaviors like excessive alcohol consumption or smoking may exacerbate both psychological distress and physical risk factors for OD.

Notably, the prevalence of chronic diseases emerged as a significant predictor of health outcomes irrespective of gender, signifying a ubiquitous health impact. Yet, the nuanced multivariate analysis among women participants accentuated the pronounced influence of comorbid conditions, suggesting a gender-disparate impact on health outcomes. Additionally, the role of psychological factors, particularly the manifestation of anhedonia, delineated notable gender-specific differences. While the association between psychological health dimensions and OD incidence was markedly significant in both univariate and multivariate analyses within the men cohort, such associations did not materialize within the female subset. This observation intimates that gender-specific mental health responses might pivotally influence the examined health outcomes, necessitating gender-tailored preventive and intervention strategies.

Our analysis revealed complex relationships between physical fitness, cognitive function, socioeconomic factors, and OD risk, with notable gender similarities and differences. T-tests revealed that individuals without OD, regardless of gender, had significantly higher income levels (*p* < 0.001), fewer comorbidities (*p* = 0.01), and higher SPPB scores (*p* < 0.001) compared to those who developed OD. Our findings both substantiate and extend the corpus of existing literature on the multifaceted etiology underpinning OD, highlighting the interplay of sociodemographic, physical, and mental health determinants (32). The delineation of both low and high BMI as pivotal indicators underscores the dual threats posed by malnutrition and obesity. Malnutrition, through its debilitative impact on pharyngeal muscle strength, directly impinges on the swallowing mechanism (33), whereas obesity predisposes individuals to conditions such as gastroesophageal reflux disease (GERD), a known precipitant of swallowing disorders (34).

Furthermore, the correlation between physical fitness, as assessed by the SPPB, and OD risk accentuates the criticality of maintaining robust physical health in ameliorating OD risk, spotlighting the essential role of muscle strength and mobility in effective deglutition (35). The SPPB score, a measure of physical fitness, showed a significant inverse relationship with OD risk in univariate analyses for both men and women. This finding underscores the complexity of OD etiology and the need for a multifaceted approach to prevention and management (35). Concurrently, the association of lower income levels with increased OD incidence highlights the intertwined challenges of healthcare accessibility, nutritional adequacy, and stress, elucidating a comprehensive framework for understanding and mitigating OD risk among older adults (36).

Cognitive impairment, with a particular emphasis on memory deficits, emerged as a salient predictive marker for one-set OD. The decrement in cognitive faculties is posited to compromise the coordination and safety of deglutition, thereby amplifying the susceptibility to aspiration and ancillary complications endemic to swallowing disorders (37). This underscores the imperative of integrating cognitive evaluations within the therapeutic framework for dysphagia management. It is worth noting that active participation in physical activities, especially outdoor activities (38), is closely related to the physical function (39) and cognition (40) of older adults, and should be identified as a protective factor for OD. Moreover, the investigation delineated a pronounced vulnerability to OD precipitated by negative affective states—specifically anhedonia—among the male geriatric cohort, a phenomenon less pronounced in their female counterparts. Chronic emotional distress is conjectured to catalyze a cascade of somatic and psychological responses, inclusive of detrimental effects on neurological integrity. The protracted ramifications of emotional stress may engender cognitive deterioration in older adults (41), potentially disrupting the neural circuits underpinning swallowing coordination (42). Additionally, the adverse emotional states are conjectured to modulate individual behavioral paradigms, such as dietary preferences and consumption patterns, further impinging upon swallowing functionality. For instance, depressive affective states may culminate in diminished appetite or disregard for nutritional adequacy, thereby exacerbating dysphagic conditions (43).

The findings from this research advocate for the adoption of a comprehensive, gender-specific approach to the early intervention and prophylaxis of one-set OD in the aging demographics. The institution of regular cognitive evaluations and rehabilitative measures is paramount for bolstering swallowing integrity and efficiency, particularly among individuals exhibiting cognitive impairment. Given the tangible impact of negative affective states on physiological well-being and deglutition dynamics, the provision of psychological assessments, emotional support mechanisms, and stress mitigation strategies is deemed essential. Behavioral and lifestyle modifications, encompassing nutritional guidance and the advocacy of salubrious eating behaviors, constitute pivotal components in the dysphagia management spectrum. For the male contingent, therapeutic strategies should particularly accentuate psychological welfare and cognitive diagnostic evaluations, reflecting their heightened susceptibility to emotion-driven OD risks. Conversely, for the female cohort, the emphasis should pivot toward physical health optimization and comorbidity management. Additionally, the sensitization of caregivers and the broader community regarding the significance of early symptomatic recognition and holistic health stewardship is crucial for the timely identification and efficacious management of OD among the aging populace.

In this investigation, leveraging data spanning from 2011 to 2018, we prognosticated the incidence of new cases of OD within a triennial framework. While the forecasting methodology employed was rigorous, it was not without its limitations. The reliance on self-reported diagnoses, as opposed to the utilization of direct clinical diagnostic methodologies such as Video fluoroscopic Swallow Study (VFSS) or Fiberoptic Endoscopic Evaluation of Swallowing (FEES), introduces potential inaccuracies stemming from subjective biases. Furthermore, NHATS dataset’s paucity of clinical measurements, including fluid analyses or imaging assessments, constrained the analytical depth, possibly eclipsing intricate facets of dysphagia’s etiology. Additionally, the dataset lacked comprehensive information on certain diseases highly relevant to OD, such as amyotrophic lateral sclerosis (ALS) and gastroesophageal reflux disease (GERD), which could have provided valuable insights into the condition’s development and progression. Moreover, the study’s demographic skew toward a predominantly non-Hispanic white cohort may circumscribe the extrapolation of these findings to more heterogenous populations. Nonetheless, a pivotal strength of this research lies in its longitudinal design, which meticulously tracks temporal changes over a three-year period, offering invaluable insights into the evolution and determinants of OD. This longitudinal perspective significantly augments the study’s contribution toward elucidating the dynamic trajectories of OD among older adults.

Looking forward, it is imperative for future inquiries to embrace a more inclusive demographic representation and to integrate clinical assessments of dysphagia. Specifically, future studies should consider employing objective diagnostic methods such as VFSS or FEES to validate self-reported results and reduce potential biases. Additionally, expanding the diversity of the sample through targeted recruitment strategies would enhance the generalizability of findings across different populations. Such enhancements will not only refine the precision of one-set OD diagnoses but also enrich our comprehension of its underpinnings, facilitating the development of more effective interventions and management strategies for this condition among aging populations.

## Conclusion

In conclusion, this study illuminates the profound impact of one-set OD on older adults’ quality of life and highlights the critical need for proactive prevention and timely intervention. Our analysis, employing random forest machine learning, has advanced the prediction of OD onset within a three-year timeframe, revealing complex interactions among socioeconomic, physical, cognitive, and emotional factors. Notably, we uncovered gender-specific risk patterns, with men showing greater vulnerability to cognitive and emotional factors, while women’s risk is more closely tied to overall health and comorbidities. These findings underscore the necessity for gender-tailored approaches in OD management. We recommend that clinical applications focus on cognitive stimulation and emotional support for men, and comprehensive health management for women. Implementation strategies should include integrating gender-specific OD risk assessments into routine geriatric care, conducting targeted community screening programs, and launching public health campaigns to raise awareness. By adopting these personalized, evidence-based approaches, we can significantly enhance OD prevention and management, ultimately improving the quality of life for diverse older adult populations.

## Data Availability

Publicly available datasets were analyzed in this study. This data can be found at: https://www.nhats.org/researcher/nhats.
